# Making the Case for Advancing Age Inclusivity

**DOI:** 10.1093/geroni/igab046.862

**Published:** 2021-12-17

**Authors:** Nancy Morrow-Howell, John Schumacher

**Affiliations:** 1 Washington University in St. Louis, Saint Louis, Missouri, United States; 2 University of Maryland, Batlimore County, Baltimore, Maryland, United States

## Abstract

How do you present the most effective case for promoting age-inclusivity to your campus leadership? Educational institutions differ in their missions and resources; and these factors affect their readiness to becoming more age-inclusive. This presentation suggests that the best approaches are tailored to intentionally and robustly advance your institution’s values, mission, and strategic plan as demonstrated through your proposed age-inclusivity initiative. We review the persuasive arguments for increasing the focus on age-inclusivity in higher education, including securing increasing external research and development funding, supporting employees and alums exploration of encore careers, attracting more students in light of demographic shifts, contributions to overall campus diversity, etc. Identifying the most compelling arguments for particular institutions, consistent with their missions, is connected to the various resources in the AFU toolkit. Finally, we show examples from a range of institutions who successfully made their cases for embracing age inclusivity and have not looked back.

